# *Sprouty4*, an FGF Inhibitor, Displays Cyclic Gene Expression under the Control of the Notch Segmentation Clock in the Mouse PSM

**DOI:** 10.1371/journal.pone.0005603

**Published:** 2009-05-19

**Authors:** Shinichi Hayashi, Taiju Shimoda, Masato Nakajima, Yuki Tsukada, Yuichi Sakumura, J. Kim Dale, Miguel Maroto, Kenji Kohno, Takaaki Matsui, Yasumasa Bessho

**Affiliations:** 1 Laboratory of Gene Regulation Research, Graduate School of Biological Sciences, Nara Institute of Science and Technology (NAIST), Ikoma, Japan; 2 Laboratory for Systems Biology, Graduate School of Information Science, Nara Institute of Science and Technology (NAIST), Ikoma, Japan; 3 Laboratory of Molecular and Cell Genetics, Graduate School of Biological Sciences, Nara Institute of Science and Technology (NAIST), Ikoma, Japan; 4 Institute for Bioinformatics Research and Development (BIRD), Japan Science and Technology Agency (JST), Tokyo, Japan; 5 Division of Cell and Developmental Biology, College of Life Sciences, University of Dundee, Dundee, United Kingdom; Ecole Normale Supérieure de Lyon, France

## Abstract

**Background:**

During vertebrate embryogenesis, somites are generated at regular intervals, the temporal and spatial periodicity of which is governed by a gradient of fibroblast growth factor (FGF) and/or Wnt signaling activity in the presomitic mesoderm (PSM) in conjunction with oscillations of gene expression of components of the Notch, Wnt and FGF signaling pathways.

**Principal Findings:**

Here, we show that the expression of *Sprouty4*, which encodes an FGF inhibitor, oscillates in 2-h cycles in the mouse PSM in synchrony with other oscillating genes from the Notch signaling pathway, such as *lunatic fringe*. *Sprouty4* does not oscillate in *Hes7*-null mutant mouse embryos, and Hes7 can inhibit FGF-induced transcriptional activity of the *Sprouty4* promoter.

**Conclusions:**

Thus, periodic expression of *Sprouty4* is controlled by the Notch segmentation clock and may work as a mediator that links the temporal periodicity of clock gene oscillations with the spatial periodicity of boundary formation which is regulated by the gradient of FGF/Wnt activity.

## Introduction

During vertebrate development, the most prominent metameric structures are the somites, which give rise to the vertebrae, the ribs, the skeletal muscles and the dermis [1]. Somites are generated as epithelial spheres of cells that sequentially bud off at regular intervals from the anterior extremity of the presomitic mesoderm (PSM) in an anterior-to-posterior direction with a strict periodicity, which is controlled by the segmentation clock [2]. The first evidence of an oscillator coupled to somite segmentation was provided by the finding of the oscillatory expression of the basic helix–loop–helix (bHLH) gene *c-hairy1* in the chick PSM [3]. The expression of *c-hairy1* oscillates in a synchronous manner among neighboring cells of the chick PSM, where the expression displays cyclic wave-like propagation patterns in a caudal-to-rostral direction by gradual phase delay. It has since been shown that several genes exhibit such a cyclic behavior in a variety of vertebrate species, including fish, chick and mouse, and some of them are evolutionarily conserved among the species [4]. Among the clock genes identified from the Notch pathway, the *hairy* related or homologous genes in mouse and fish, namely *Hes* and *her*, also cycle [5]–[9]. However, cyclic PSM expression of *lunatic fringe* (*Lfng*), which encodes a glycosyl-transferase that modulates activity of the Notch receptor, is detected in mouse and chick [10]–[12], but not in fish [13], [14]. Some genes in the Wnt and FGF pathway display oscillatory gene expression in the mouse PSM, such as *Axin2* and *Nkd1*, both inhibitors of Wnt signaling, *Sprouty2* and *Dusp4*, both inhibitors of FGF signaling, and *Snail1*, a transcriptional repressor [15]–[19], but cyclic behavior of their homologues in other vertebrate species have not been reported, other than *Snail2* in chick.

In the PSM, a complex gene network that includes several feedback loops could elicit highly dynamic gene expression to form the robust segmentation clock. In mouse, oscillating Hes7 represses *Lfng* and its own transcription periodically and establishes a feedback loop, which is essential for cyclic gene expression and participates in the mechanism of the segmentation clock [20]. Lfng modulates Notch activity periodically and forms a negative feedback loop, which provides cyclic Notch activity in the chick PSM [21]. A negative feedback of Axin2 also generates cyclic Wnt signaling in mouse PSM, which is essential for somite formation [17].

FGF and Wnt signaling are both crucial to determine the position of somite boundary specification [17], [22], [23]. Both the FGF8 and Wnt3A ligands establish posterior-to-anterior gradients of expression in the PSM [24]. The position of the determination front demarcates the region where the PSM cells are able to embark on their segmentation program and the temporal periodicity of oscillatory gene expression becomes converted to the spatial periodicity of the somites. Several levels of crosstalk between these pathways and the segmentation clock have been reported. Thus, FGF signaling initiates the oscillation of *Hes7* in the mouse PSM [18]. An FGF downstream gene, *her13.2* is required for the auto-repression of *her*, which is the key mechanism of the segmentation clock in zebrafish [25]. However, it largely remains to be elucidated how the segmentation clock cooperates with the Wnt and FGF pathways to generate regular interval pattern.

We have shown that the FGF target and negative regulator, *Sprouty4*, shows oscillatory gene expression in the mouse PSM. *Sprouty4* cycles in phase with other Notch regulated clock genes, such as *Lfng* and *Hes7*, and its oscillation depends on Hes7, which is one of the core factors of the mouse segmentation clock mechanism. Thus, *Sprouty4* could be one of the candidates for the mediator that integrates spatiotemporal information in somitogenesis. We further find the cyclic expression of *Sprouty4* is not evolutionarily conserved since it does not oscillate in the zebrafish PSM.

## Results and Discussion

### The expression of *Sprouty4* oscillates in the mouse PSM

**Figure 1:**
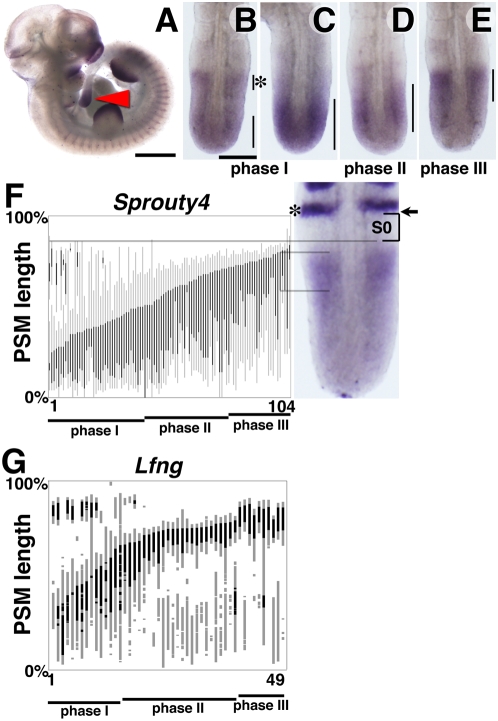
Cyclic expression pattern of *Sprouty4* in mouse PSM. (A–E) Expression pattern of *Sprouty4* at E10.5. *Sprouty4* mRNA is expressed in the caudal region of somites, limb buds, and frontonasal processes as well as the PSM (red arrowhead) (A), in which various expression patterns were observed, categorized as phase I (B,C), II (D) and III (E). (F,G) Analysis of *Sprouty4* and *Lfng* expression in the mouse PSM. Each vertical bar represents one embryo. Black stripes represent stronger expression domains as compared to gray bars. We examined 104 embryos for *Sprouty4* (E), and 49 embryos for *Lfng* (F). We used *Uncx4.1* probe as a positional marker (asterisk in E). The position of S0 (bracket), the forming somite, and the boundary of the newest somite (arrow) are indicated, respectively. Scale bars: A, 1 mm; B, 200 µm.

The mRNA expression of *Sprouty4* coincides with regions of FGF signaling activity in the mouse embryo at embryonic day (E) 10.5, including the PSM, the somites, the limb buds and the frontonasal processes as previously reported (Figure 1A) [26], [27]. Among a group of stage matched E10.5 embryos, the expression pattern of *Sprouty4* in the PSM varied considerably. The expression patterns can be grouped into 3 phases [28]. In some embryos, the expression domain extends throughout the posterior PSM and tail bud region (Figure 1B,C, *n* = 42/104, phase I). The second group of embryos displayed a broad pattern of expression with the strongest signal in the middle part of the PSM (Figure 1D, *n* = 36/104, phase II). The last group of embryos showed the highest intensity of expression in the anterior part of the PSM with no expression in the posterior PSM and tail bud region (Figure 1E, *n* = 26/104, phase III). In order to more precisely determine the dynamic expression profile of *Sprouty4* in the PSM, we measured the domains of *Sprouty4* expression in the PSM of individual embryos, scored the distance between the boundary of the newest somite and the anterior limit of PSM expression and represented these measurements graphically in order of increasing length of expression domain from the posterior end of the PSM (Figure 1F) [21]. The stacked expression patterns of embryos (*n* = 104) indicate that *Sprouty4* mRNA expression changes as a continuous progressive wave from the posterior to the anterior end of the PSM. This expression profile is similar to that of other cyclic genes such as *Lfng* in the mouse PSM (Figure 1G) and *Lfng*, *c-hairy1*, *c-hairy2* in the chick PSM [21], [29]. The only difference is that while the rate of progression of the wave of expression of these other clock genes is much faster in the posterior than the anterior PSM, that of *Sprouty4* appears uniform along the whole length of the mouse PSM (Figure 1F).

Some embryos in phase I, in addition to the caudal expression domain also display a narrow band of expression in the anterior part of the PSM (Figure 1B, asterisk), which most likely corresponds to the domain of expression in phase III, suggesting the expression of *Sprouty4* in the mouse PSM may be cyclic. We found that 42, 35 and 29 embryos display Phase I, II and III, respectively and thus the ratio of embryo number in each phase indicates a roughly equal distribution in each phase which suggests each phase lasts roughly an equivalent time. In the case of *Lfng*, 15, 24 and 10 embryos were classified as Phase I, II and III, respectively (*n* = 49). These results suggest that the expression of *Sprouty4* is cyclic and displays similar pattern to that of *Lfng* in the mouse PSM.

### The expression of *Sprouty4* oscillates in 2-h cycles in phase with the Notch regulated cyclic gene, *Lfng*

**Figure 2:**
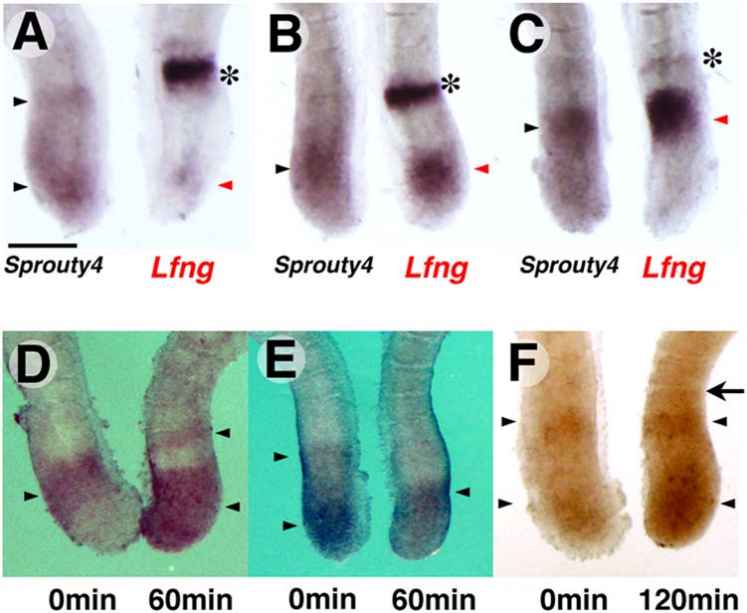
The expression of *Sprouty4* oscillates in 2-h cycles in phase with *Lfng.* (A–C) Comparison of the expression of *Sprouty4* (left) and *Lfng* (right) in bisected caudal portions of E10.5 mouse embryos. The expression patterns are categorized into three phases: phase I (A), phase II (B) and phase III (C). The expression of *Sprouty4* (black arrowhead) is similar to that of *Lfng* (red arrowhead) in each phase, except the *Sprouty4* profile does not include the anterior band of the *Lfng* profile (asterisks). (D–F) The caudal portions of E10.5 mouse embryos were bisected. One half (left) was fixed immediately, and the other half (right) was cultured for 60 min (D,E) or 120 min (F), before fixation. The expression patterns of *Sprouty4* (arrowheads) of the cultured halves were different from the other halves when they were cultured for 60 min. However, when the cultured halves were incubated for 120 min, the patterns were similar to the control halves and they had a new boundary (arrow). Scale bar: A, 200 µm.

Previous reports clearly demonstrated that, in the mouse PSM, the expression of *Lfng* shows a cyclic pattern, which coincides with the periodicity of somite formation [11], [12], and that oscillating genes belonging to the Notch and FGF pathways cycle synchronously [19]. However, some cyclic genes in the Wnt pathway, including *Axin2,* oscillate out of phase with the Notch clock genes [17], [19]. To investigate whether the expression of *Sprouty4* oscillates and , if so, to investigate that it oscillates in or out of phase with *Lfng*, we bisected the posterior region of E10.5 mouse embryos and analysed *Lfng* or *Sprouty4* mRNA in respective halves of each embryo. In all embryos, both were expressed in the same regions of the PSM (*n* = 23, Figure 2A–2C), although *Sprouty4* was never expressed in the anterior most region as a narrow and sharp band, which is seen in the *Lfng* expression profile (asterisks in Figure 2A–2C).

To clarify whether the expression of *Sprouty4* oscillates further, we carried out an explant culture experiment. The posterior region of embryos was bisected and one half was fixed immediately and the other half was cultured for 60 min or 120 min before fixation. When one half was cultured for 60 min, the expression patterns of *Sprouty4* were different from those of the uncultured half (Figure 2D,E, *n* = 10). By contrast, when we cultured one half for 120 min, the expression patterns of *Sprouty4* were very similar to those of uncultured half (Figure 2F, *n* = 4), and we observed a newly formed boundary in the explants (Figure 2F, arrow). Taken together, these results indicate that the expression of *Sprouty4* oscillates in 2-h cycles in the mouse PSM in synchrony with the Notch regulated clock genes.

### Hes7 controls the cyclic expression of *Sprouty4* via transcriptional inhibition

**Figure 3:**
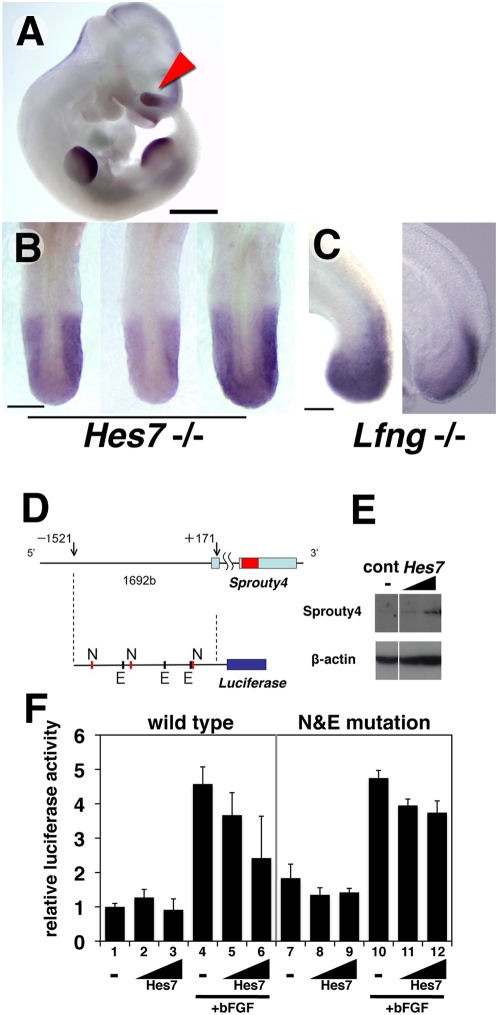
Cyclic expression of *Sprouty4* depends on *Hes7.* (A–C) Expression pattern of *Sprouty4* in *Hes7*-null or *Lfng*-null mutant embryos. In E10.5 *Hes7*-/- mutant embryos, *Sprouty4* is expressed in similar tissues as seen in wild-type embryos (A). All *Hes7*-/- mutant embryos display a uniform expression in the PSM (*n* = 24) (B). *Lfng*-/- mutant embryos display various expression patterns in the PSM (*n* = 5) (C). Scale bars: A, 1 mm; B, 200 µm; C, 100 µm. (D–F) Promoter analysis of *Sprouty4*. (D) The 1.7 kb *Sprouty4* promoter, which contains 3 consensus sequences of N-box and 3 consensus sequences of E-box, was inserted upstream of *luciferase*. (E) Hes7 protein was specifically detected by Western blotting with anti-Hes7 antibody in the *Hes7* transfected cells. β-actin were detected in Hes7 transfected and control cells. (F) Luciferase activity under the control of the *Sprouty4* promoter was not significantly suppressed in the presence of Hes7 (lane 2,3). However, the activity induced by bFGF (lane 4) was inhibited in the presence of Hes7 (lane 5,6). Luciferase activity under the control of the *Sprouty4* promoter with mutations of N-box and E-box was not significantly suppressed in the presence of Hes7 again (lane 8,9). The activity induced by bFGF (lane 10) was not significantly inhibited in the presence of Hes7 (lane 11,12).

The cyclic expression of *Lfng*, as well as that of *Nkd1* and *Hes7* itself, is controlled by Hes7 via periodic transcriptional repression [15], [20]. In order to examine whether the cyclic expression of *Sprouty4* is also under the control of Hes7, we analysed *Sprouty4* mRNA expression in the absence of Hes7 [5]. The expression of *Sprouty4* is not affected in *Hes7*-null mutant mouse embryos in tissues other than the PSM, although the metameric pattern in the somites is faded (Figure 3A). This result is consistent with the previous observations, in which the expression pattern of *Hes7* is restricted to the PSM [30] and the phenotype of *Hes7*-null mutant mice embryos whose somites are severely disorganized [5]. In all mutants examined (*n* = 24), *Sprouty4* is uniformly expressed throughout the PSM (Figure 3B). Interestingly, although the phenotype of *Lfng*-null mutant mice closely resembles that of *Hes7*-null mice in somitogenesis [31], [32], *Sprouty4* still oscillates in the PSM of *Lfng*-null mutant mice (Figure 3C). Thus, the cyclic expression of *Sprouty4* depends on *Hes7*.

Since Hes7 works as a transcriptional repressor that binds to E-box and N-box sequences [30] and transcription of *Lfng* and *Hes7* are periodically repressed by Hes7 to generate their oscillatory gene expression profile [20], we next examined whether Hes7 can inhibit the expression of *Sprouty4* by a promoter analysis. Because the 1.7 kb upstream region of human *Sprouty4* is sufficient for the transcription in response to FGF signaling[33], we took the 1.7 kb promoter region of mouse *Sprouty4* upstream of a *luciferase* reporter gene (Figure 3D). When we transfected the reporter carrying the *Sprouty4* promoter into the mouse cell line, the stimulation with basic FGF (bFGF) increased the reporter activitiy (Figure 3F, left), suggesting that the mouse *Sprouty4* promoter, which we used here could response to bFGF, similar to the human promoter [33]. When we co-transfected *Hes7* with the reporter (Figure 3E), Hes7 could suppress the bFGF-dependent transcriptional activity, but did not inhibit the basal level of the promoter activity (Figure 3F, left). This observation is consistent with previous results showing Hes7 inhibits *Hes7* promoter activity induced by Notch signaling but it does not effectively inhibit the baseline activity of the *Hes7* promoter [20], which is periodically suppressed by Hes7 itself in the mouse PSM. Since the promoter region contains several *Hes7* responsive elements, E-box and N-box consensus sequences [30], we generated the *Sprouty4* promoter without E-box and N-box sequences by multiple mutagenesis [34], [35]. Although the *Sprouty4* promoter without E-box and N-box was normally activated by bFGF, the inhibition of bFGF-dependent activation by Hes7 was partially canceled (Figure 3F, right), suggesting that E-box and N-box sequences act as the Hes7-responsive elements. Thus, *in vitro* situation, Hes7 can inhibit the expression of *Sprouty4* through the Hes7-responsive elements in the *Sprouty4* promoter.

**Figure 4:**
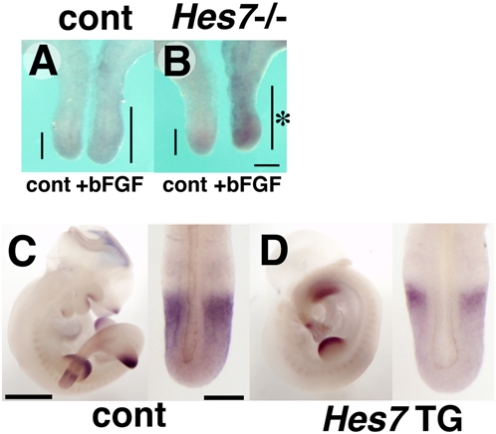
Hes7 can inhibit the expression of *Sprouty4* in the PSM. (A,B) Explant culture with *Hes7*-null embryo. (A) The expression of *Sprouty4* (bars) was slightly induced by 4-h exposure of bFGF in the wild-type PSM. (B) In the PSM of *Hes7*-null embryos, 4-h exposure of bFGF dramatically induced *Sprouty4* expression in the PSM (asterisk). (C,D) Over expressed *Hes7* inhibits the expression of *Sprouty4* in the PSM. The expression of *Sprouty4* was detected in the transgenic embryos overexpressing *Hes7* from the 5 kb *Hes7* promoter (D) and wild-type embryos (C). In the PSM of transgenic embryos, the expression of *Sprouty4* was exclusively down-regulated. Scale bars: B, 200 µm; C, 1mm (left), 200 µm (right).

To examine whether Hes7 inhibits Sprouty4 expression *in vivo*, we performed two experiments: an explant culture of the PSM of *Hes7*-null mutant embryos and an overexpression of *Hes7* in the PSM by transient transgenic analysis. In former experiments, we bisected the posterior region of *Hes7*-null or wild-type embryo, and treated with bFGF for 4h only in one half. In the PSM of wild-type embryo, which normally expresses *Hes7*, treatment of bFGF slightly induced *Sprouty4* expression (Figure 4A, *n* = 10). However, in the PSM of *Hes7*-null mutant, treatment of bFGF dramatically increased *Sprouty4* expression (Figure 4B, *n* = 3). Conversely, in the latter experiments, overexpression of *Hes7* specifically in the PSM by using *Hes7* transgenic mice inhibited *Sprouty4* expression just in the PSM (Figure 4D, *n* = 6/10), compared to the transgene-negative littermates (Figure 4C, *n* = 11), although both showed similar patterns and levels of *Sprouty4* expression in tissue other than the PSM. These data indicate that Hes7 can inhibit *Sprouty4* expression in the PSM. Taken together, it is likely that cyclic Hes7 inhibits the *Sprouty4* promoter periodically in the mouse PSM, resulting in the oscillatory expression profile of *Sprouty4*.

### Cyclic expression of *Sprouty4* is not observed in the zebrafish PSM

**Figure 5:**
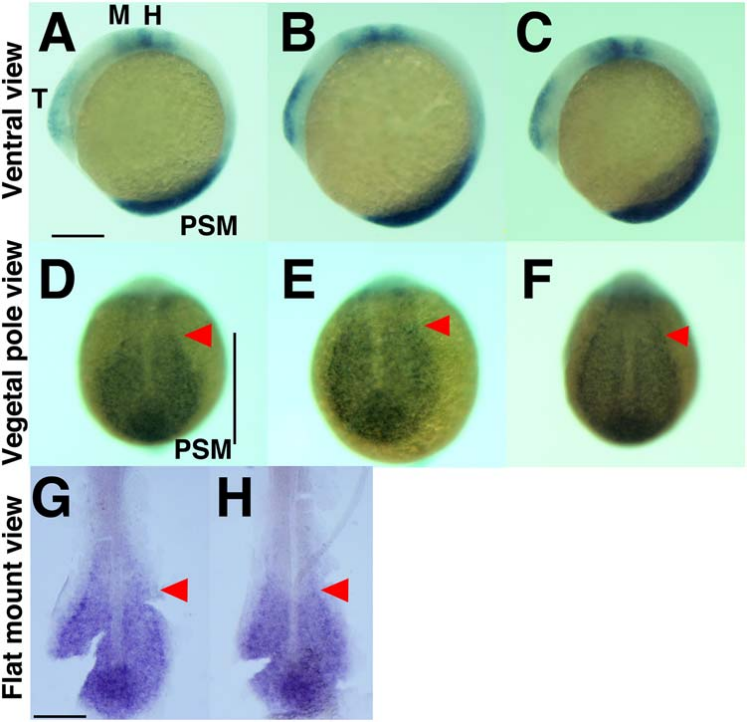
*Sprouty4* is expressed in zebrafish PSM but not cyclic. (A–C) Whole mount embryos show expression of *Sprouty4* in the PSM, telencephalon T), midbrain-hindbrain boundary (M), heart and brachial arch primordia (H) in lateral views of 6 somite stage zebrafish embryos. Lateral view and anterior to the top. (D–F) The expression patterns of *Sprouty4* in the zebrafish PSM do not vary among embryos. Dorsal view and anterior to the top. The stained embryos were flat mounted by removing the yolk (G, H). Red arrowheads indicate the anterior limit of the *Sprouty4* positive region. Scale bars: A, G, 200 µm.

Finally, we examined the expression profile of the orthologue of *Sprouty4* in the zebrafish PSM. Some genes in the Notch pathway including *her1*, *her7* and *deltaC* show cyclic expression in the zebrafish [6]–[8], [36], but other genes which cycle in the mouse PSM do not display cyclic expression or have not been well examined in zebrafish [14]. These observations mean that the cyclic expression of some but not all genes is conserved among species. In zebrafish, *Sprouty4* is expressed in the region of FGF signaling, including the PSM, the midbrain-hindbrain boundary, the heart and the brachial arch primordia at the 10 somite stage (Figure 5A–5C). This expression pattern is highly conserved with that of mouse (Figure 1A) [26], [27]. However, we did not find evidence of any cyclic expression of *Sprouty4* in the zebrafish PSM (*n* = 12) (Figure 5D–5H). These results suggest that the oscillatory behavior of *Sprouty4* in the mouse is not conserved in zebrafish.

### Sprouty4 is a strong candidate for the mediator that integrates spatiotemporal information during somitogenesis

**Figure 6:**
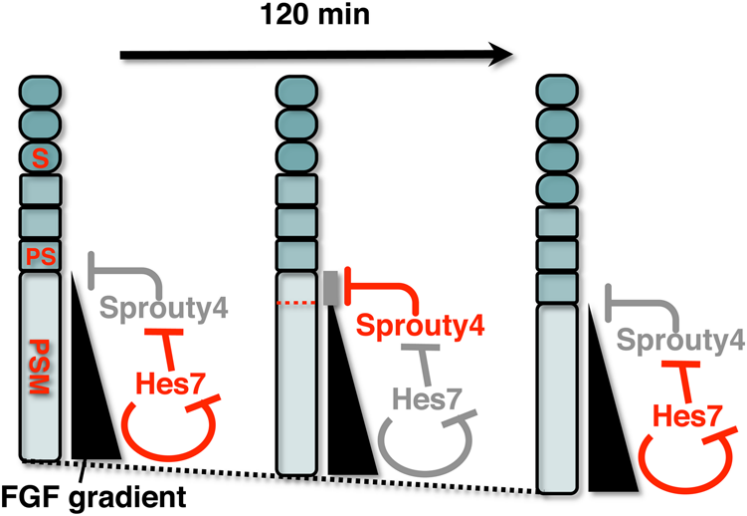
A Putative Model for the role of Sprouty4 as a mediator that links the segmentation clock to the gradient of FGF signaling. In the PSM, FGF signaling establishes a posterior-to-anterior gradient, which is involved in the positioning of presumptive somite boundaries. Cyclic Sprouty4, which is controlled by the Notch segmentation clock, the mechanism of which includes negative feedback loop of Hes7, may inhibit the FGF signaling possibly around the anterior border of the FGF signaling positive area, where the FGF signaling is close to its threshold. Thus, the FGF signaling may be periodically inhibited by Sprouty4, by which temporal periodicity of Notch segmentation clock may be translated to spatial periodicity of the array of somites. S, somite; PS, presumptive somite.

In the PSM, FGF signaling establishes a posterior-to-anterior gradient, which is involved in the positioning of somite boundaries [22], [23]. Although the precise target is unclear, Sprouty4 inhibits the FGFsignaling [37], [38]. Our results in this study revealed that the expression of *Sprouty4* oscillates in phase with *Lfng* and hence also with *Hes7* in the mouse PSM, and that this cyclic expression depends on *Hes7*. Thus, the periodic expression of Sprouty4 potentially acts to periodically repress FGF signaling and thereby periodically modulate spatial information in the mouse PSM (Figure 6). Therefore, we propose that Sprouty4 could be one of the candidates for the mediator that integrates spatiotemporal information during somitogenesis. Of course, other feedback inhibitors that show cyclic expression in the PSM, including Sprouty2 and Dusp4, are also candidates for this putative mediator activity. Most probably, these factors work together redundantly during somitogenesis, since for example, no obvious abnormalities in somite segmentation have been reported in the null mutant of *Sprouty2* [39], [40] or *Sprouty4* [41], or in the double mutant of the two genes [42], although a detailed investigation into the segmental patterns in these mutants is still required. FGF inhibitors, acting as putative mediators, may periodically suppress FGF signaling in the middle to anterior PSM around the position of the determination front in order to generate a discrete pattern, by which the position of somite boundaries is determined.

## Materials and Methods

### *In Situ* Hybridization and Graphical Analysis

Whole mount *in situ* hybridization were carried out as previously described[30], [43]. The following regions were used as probes; mouse *Sprouty4*, nucleotide residues -25-1177; *Lfng*, 17-1382; *Uncx4.1*, -14-1680, zebrafish *Sprouty4*, 32-830. We detected expression of *Sprouty4* or *Lfng* in E10.5 mouse PSM by BCIP/NBT. The expression pattern was digitally scored Sion image software. The relative positions in the PSM were normalized by the lengths between the newest somite boundary (100%), for which we used *Uncx4.1* probe as a marker, and posterior end of embryo (0%). The values from each assessment were analyzed graphically.

Experiments were approved by the Animal Care Committee of Nara Institute of Science and Technology and were conducted in accordance with guidelines established by the Science Council of Japan.

### Explant Culture

The posterior part of embryo was bisected at E10.5. One half was fixed immediately, and the other half was cultured in 10% FBS-DMEM/F12 for 60 or 120 min before fixation. *Hes7*-null mutants and its littermates were bisected and cultured for 4 h in 1% FBS-DMEM/F12 with or without bFGF (20 nanogram/ml). After fixation, the expression of Sprouty4 was detected by in situ hybridization.

### Transgenic mice

For transient transgenic experiment, we used a vector containing the 5.4 kb *Hes7* promoter followed by exonic regions of *Hes7* with IRES-Venus and SV40 polyadenylation signal [18].

### Luciferase Assays

For promoter analysis, a luciferase reporter (GL3, Promega, 50 nanogram) under the control of the *Sprouty4* promoter (−1521 to +171) were transfected into NIH3T3 cells, which were plated in 24-well plates, with 0, 25, 50 nanogram of expression vector (pCI, Promega) for Hes7 as previously described[30]. The vector for Renilla luciferase gene under the control of the SV40 promoter (5 nanogram) was co-transfected as an internal standard to normalize the transfected efficacy. After 24 h, bFGF (10 nanogram/ml) were added to the culture media and incubate for 24 h. Then, the cells were harvested and luciferase activities were measured. To introduce mutations to the promoter of Sprouty4, site-directed and semi-random mutagenesis was performed as previously described[44]. Primer sequences (underlines show mutated nucleotide). N-box1 Mutant: CTATGAAGGCCAAACCATGGCAAGATAGATCTATC, E-box1 Mutant: CTGCTCCACCCATCTGCTCAGCTCATTCTCCCTAT, N-box2 Mutant: AAAGGGGAGAGGGCCCATGGAATACAAAGGCCTGG, E-box2 Mutant: CCACGCAGCTAAGCTGGTCACTGCAGTCGCCGCCG, E-box3 and N-box3 Mutant: GCGCGCACGGGGTTGGTCGACCCCACCCATTCATA.

### Western Blotting

NIH 3T3 cells were transfected with 0, 25, 50 nanogram of *Hes7* expression vector. After 48 hour, they were lysed in lysis buffer (50 mM Tris pH 8.0, 300 mM NaCl, 2 mM EDTA, 1%NP-40, 0.1% SDS and 0.5% Deoxycolate). The lysates were sonicated for 30second interval and 10second and it was repeated five times, and were separated by SDS-PAGE and transferred to polyvinylidene difluoride membranes. Then, the membrane was incubated with 1/500 anti-Hes7 antibody[20], followed by applying anti-guinea pig IgG antibody conjugated peroxidase.

### Zebrafish

Wild type (AB) was used in this work.
